# Risk factors of lymphatic filariasis in Asia: a systematic review

**DOI:** 10.1186/s12879-025-12158-w

**Published:** 2025-12-04

**Authors:** Lahiru Sandaruwan Galgamuwa, Navodi Mekala Hakmanage, Sara Fernando

**Affiliations:** 1 Department of Parasitology, Faculty of Medicine, Sabaragamuwa University of Sri Lanka, Ratnapura, Sri Lanka; 2 Department of Computer Systems Engineering, Faculty of Computing and Technology, University of Kelaniya, Kelaniya, Sri Lanka

**Keywords:** Lymphatic filariasis, Asia, Risk factors

## Abstract

**Background:**

Lymphatic filariasis remains a major public health challenge across many parts of Asia, where environmental, socioeconomic, behavioral, and programmatic factors converge to sustain transmission. Understanding the risk factors for lymphatic filariasis supports in guiding targeted interventions, including enhancing mass drug administration (MDA), strengthening vector control, and creating educational initiatives for the community. This focused strategy can increase the efficacy and efficiency of LF elimination initiatives in various Asian contexts.

**Methods:**

This review was conducted with available literature to assess risk factors of lymphatic filariasis in Asia in line with the PRISMA (Preferred Reporting Items for Systematic Reviews and Meta-Analyses) guidelines. Searches were performed in PubMed, Google Scholar, Science Direct and Springer Nature Link databases and included 12,402 original English-language studies published up to January 2025. A total of forty four journal articles containing factors associated with lymphatic filariasis in different microscopic and clinical diagnostic stages, including over 284,908 participants were finally assessed for this review.

**Results:**

Circulating filarial antigen (CFA) was consistently higher among males and older individuals, particularly in low-elevation areas and densely populated households in Myanmar and Sri Lanka. Factors related to occupation and socioeconomic status, such as outdoor employment, low income, and poorly constructed housing, were associated with a greater risk of infection in Malaysia and Indonesia. Similar patterns were noted for microfilaremia, with male gender, advancing age, and poorly constructed housing identified as significant predictors in various studies conducted in India and Indonesia. Other contributing factors included low educational attainment, inadequate sanitation, inconsistent use of bed nets, proximity to drainage systems, and migration status in Asia.

**Conclusion:**

The findings indicate that the transmission of lymphatic filariasis (LF) in Asia is influenced by a combination of demographic, social, and environmental elements. This underscores the necessity for comprehensive vector control and community-oriented initiatives that address both the health and social dimensions of the disease.

**Supplementary Information:**

The online version contains supplementary material available at 10.1186/s12879-025-12158-w.

## Introduction

Lymphatic filariasis (LF) is a parasitic disease caused by the filarial worms *Wuchereria bancrofti*, *Brugia malayi*, and *Brugia timori* and spread by mosquitoes [1]. LF is classified as one of the Neglected Tropical Diseases (NTDs) that can be managed through preventive chemotherapy [2]. It is predominantly found in tropical and subtropical regions, especially impacting impoverished and marginalized populations. Globally, *Wuchereria bancrofti* is responsible for approximately 91% of all LF infections [3]. The disease presents as painful and disfiguring swelling known as lymphoedema or hydrocoele, frequently resulting in long-term disability [4]. Individuals affected by lymphatic filariasis often face social stigma through isolation, judgment and rejection from the family and the community [5]. In 2023, approximately 657 million people in 39 countries where LF are at risk, with around 120 million believed to be currently infected [6].

LF continues to be a major public health problem in various Asian countries, where favorable environmental conditions and socio-economic elements persist in promoting its transmission. Active cases are still being reported in countries such as India, Indonesia, Bangladesh, Myanmar, Nepal, and the Philippines, with *Wuchereria bancrofti* and *Brugia malayi* as the primary parasites responsible [7, 8]. Additionally, small foci of *Brugia timori* infections have been identified in Indonesia [9].

Multiple complex factors contribute to filariasis, including the parasite itself, humans acting as hosts, and environmental, biological, and social conditions especially the local community’s cultural practices and economic status [10, 11]. In addition to the presence of vectors and host organisms, environmental conditions significantly influence the transmission of the disease [10, 11].

Individuals living in areas where lymphatic filariasis is widespread are at greater risk of infection, especially those with low income and regular exposure to mosquito bites. Contributing factors include inadequate sanitation, overcrowded living conditions, and occupations that require outdoor activity, such as agriculture or hunting, which increase the likelihood of mosquito contact [12]. Cultural factors also lessen the efficacy and acceptance of lymphatic filariasis prevention strategies, such as a lack of community involvement and a strong belief in individual privacy [13].

The World Health Organization (WHO) seeks to eliminate lymphatic filariasis as a public health problem by interrupting its transmission and decreasing the number of individuals affected. Sri Lanka, Thailand, Vietnam, Cambodia, and the Lao People’s Democratic Republic having been officially recognized by the WHO as having eliminated LF as a public health problem [14, 15]. However, many of these countries do not have sufficient post-elimination surveillance systems in place to identify any potential recurrence. Maintaining ongoing surveillance is crucial to preventing re-emergence, especially in regions with high infection rates in the past or where risk factors are still present [16]. In Southeast Asia, including Thailand, control measures face significant challenges because *Brugia malayi* can be spread from animals particularly cats to humans, which complicates elimination efforts due to its zoonotic characteristics [17].

Recognizing the factors that sustain or promote LF transmission is important for obtaining and maintaining elimination objectives [18]. This systematic review intends to identify and compile the various environmental, biological, and socio-demographic aspects linked to LF across Asia. The results will aid policymakers and public health officials in improving surveillance methods, focusing on high-risk groups, and creating tailored interventions to break the transmission cycle and prevent the disease from re-emerging in settings aimed at elimination.

## Materials and methods

### Protocol

This review was designed to understand behavioral, environmental, and socio demographic risk factors for lymphatic filariasis endemic countries in Asia. It is the largest continent, bordering on the Arctic Ocean, the Pacific Ocean, the Indian Ocean, and the Mediterranean and Red Seas in the west. Asia is the largest and most populous continent on Earth, covering nearly one-third of the planet’s landmass and hosting over 60% of the global population. In this systematic review, we followed the PRISMA 2020 guidelines and checklist to minimize potential bias [19].

### Search strategy

A bibliographic search was conducted for peer-reviewed articles published from the last 50 years (1975 to 2025). Only studies published in the English language were included in this review. There were no restrictions regarding participant age, sample size, study design, or statistical power. The literature search was carried out using four databases: PubMed, Google Scholar, Science direct and Springer nature Link. This review was conducted with available literature to assess risk factors of lymphatic filariasis in Asia in line with the PRISMA (Preferred Reporting Items for Systematic Reviews and Meta-Analyses) guidelines.

The search encompassed all peer-reviewed articles, regardless of national or international scope. The PubMed, Google Scholar, Science direct and Springer nature Link databases were searched using key terms such as (Risk factors or factors) AND (lymphatic filariasis or *Wuchereria bancrofti* or *Brugia timori* or *Brugia malayi* or lymphedema or hydrocele or elephantiasis or microfilariae or microfilaremia”) AND (“Myanmar or Burma or Thailand or Laos or Cambodia or Vietnam or Malaysia or Bangladesh or India or Sri Lanka or Maldives or Yemen or Nepal or Philippines or Pakistan or Indonesia).

### Study selection and data extraction

The initial selection of articles was conducted independently by two reviewers. Prior to screening, duplicate entries were removed. The remaining studies underwent a preliminary selection based on titles and abstracts. Studies deemed ineligible at this stage were excluded, and the remaining articles proceeded to full-text review. Exclusion criteria included (a) narrative or systematic reviews, with or without meta-analyses; (b) book chapters; (c) conference proceedings; (d) letters to the editor; (e) case reports; (f) studies not addressing risk factors.

From the studies that met the inclusion criteria, the following data were extracted: (a) author(s), (b) country of study, (c) identified filarial species, (d) study population, and (e) associated risk factors. Data extraction was independently performed by the two reviewers, and any discrepancies were resolved by consultation with a more experienced third reviewer. Additionally, reference lists of the selected articles were manually screened to identify any relevant studies that may have been missed during the database search. Clinical presentations were analyzed to identify risk factors associated with LF. Information regarding circulating filarial antigens, presence of microfilaria, lymphedema, hydrocele and elephantiasis conditions was also collected to gain a deeper understanding of the factors related to these various conditions.

## Results

### Database search and study selection

**Fig. 1:**
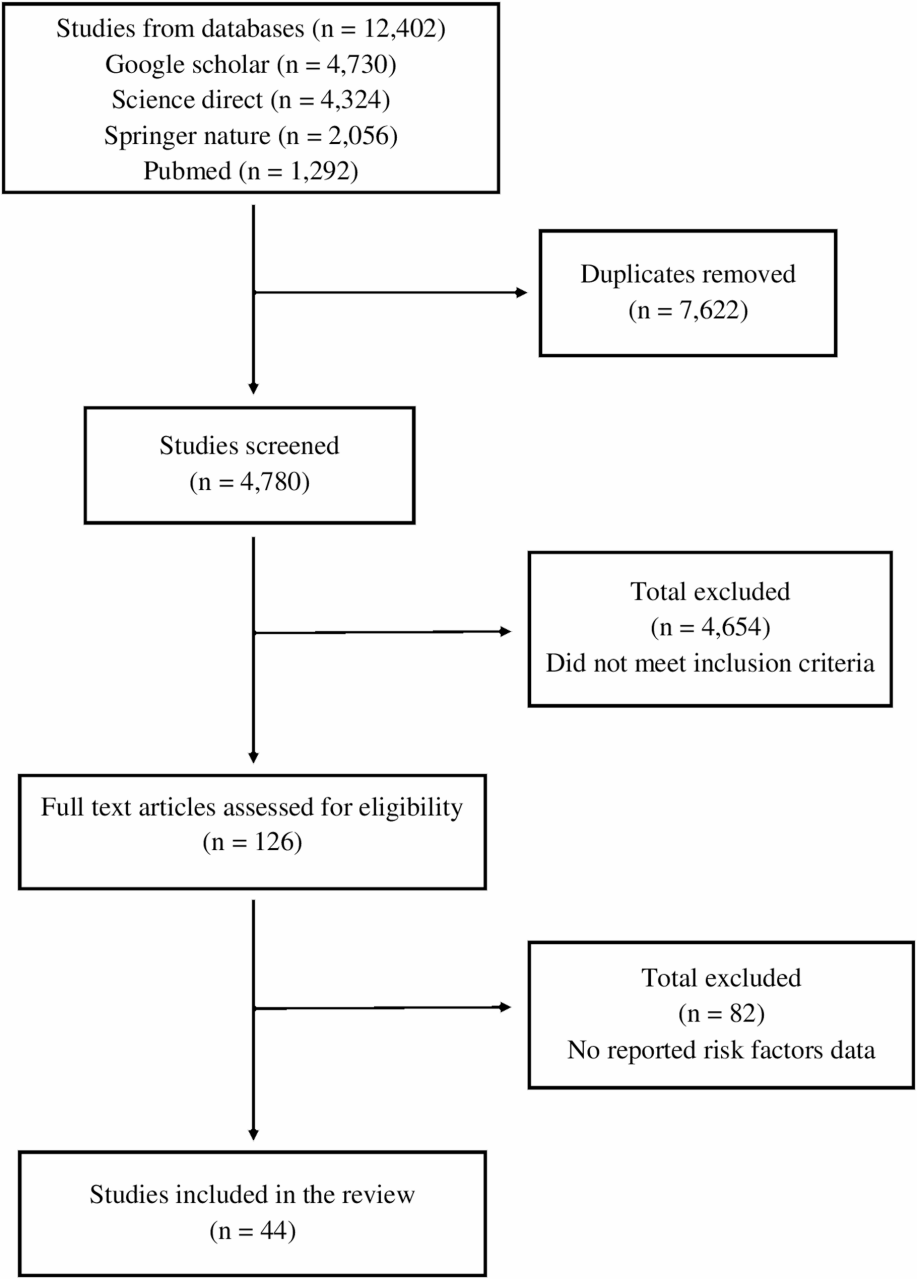
Flow diagram systematic search and review process

An initial search across four databases retrieved 12,402 records including 1,292 from PubMed, 4,730 from Google Scholar, 4324 from Science direct and 2056 from Springer nature link. After removing 7,622 duplicates, 4,780 unique records remained for title and abstract screening. This process excluded 4,654 articles, leaving 126 for full-text review. Of these, 82 were excluded because they did not fulfill all predefined inclusion criteria. As a result, 44 studies were included based on the study criteria. This process was illustrated in the PRISMA flow diagram (Fig. 1).

### Characteristics of included studies

In this review risk factors were analyzed with different clinical/microscopic diagnosis of lymphatic filariasis. A total of 10 studies detected risk factors associated with circulatory lymphatic filarial antigen. In addition, 19 studies were identified risk factors with microfilaria of lymphatic filarial worms and 28 studies based on clinical manifastations of chronic disease such as hydrocele, lymphoedema, and elephantiasis. These studies primarily focused on countries in Asia, including India (18), Sri Lanka (4), Bangladesh (1), Thailand (4), Myanmar (2), Cambodia (2), Malaysia (2), Philippines (1), Indonesia (8), Nepal (2). There were no studies included from Lao PDR, Vietnam or Yemen. Among the studies reviewed, 18 indicated the existence of *Wuchereria bancrofti*, 11 recognized *Brugia malayi*, and 2 reported the finding of both *Wuchereria bancrofti* and *Brugia malayi*. Across the studies, a total of 284,908 participants were evaluated, with individual study sample sizes ranging widely from 70 to 85,974.

Older age and being male were the most frequently identified risk factors for circulating filarial antigens (CFA) associated with Wuchereria bancrofti and Brugia malayi. Other contributing factors for CFA were residing at a low altitude, high population density in households, low socioeconomic status, occupations that require outdoor work, not usage of bed nets, and migration from areas where these infections are common. (Table 1).

**Table 1:** Risk factors associated with Circulatory Filarial Antigens (CFA) of lymphatic filarial parasites

Study (year)	Study area	Sample size	Species	Risk factors
Dickson et al., (2021) [20]	Mandalay Region, Myanmar	1014	*W.b / B.m.*	• Older age • Males • Low elevation • Density of people per household room
Dickson et al., (2018) [21]	Mandalay Region, Myanmar	1014		• Older age • Males • Amarapura Township
Chan et al., (2022) [22]	Sabah, Malaysia	10,100	*W.b. / B.m.*	*B.m.* – • Low socio-economic status • Older age *W.b.* - no
Zakaria and Avoi, (2022) [23]	Sabah, Malaysia	244	*B.m.*	• Older age • Outdoor jobs
Terhell et al., (2000) [24]	South-Sulawesi, Indonesia	171	*B.m.*	• Male
Wahyuni et al., (2004) [25]	South Sulawesi, Indonesia	583	*B.m.*	• Older age
Rao et al., (2018) [26]	Southern province, Sri Lanka	528	*W.b.*	• Male
Rao et al., (2014) [27]	Southern, Western and North-Western provinces, Sri Lanka	30,833		• Male • Older age
Rao et al., (2017) [28]	Southern and Western province, Sri Lanka	5400	*W.b.*	• Male • Not usage of bed nets
Bhumiratana et al., (2002) [29]	Tak Province, Thailand	219	*W.b.*	• Migrants from Myanmar

Wb: *Wuchereria bancrofti*; Bm: *Brugia malayi*

When considering microfilaremia, male gender and older age were the most commonly identified risk factors for both *Wuchereria bancrofti* and *Brugia malayi* infections. Other contributing factors differed by region and included a history of mosquito bites, low household income, lower levels of education, poorly built housing, closeness to drainage systems, residing in sub-coastal or coal-mining regions, minimal clothing, inconsistent or non-use of bed nets, inadequate sanitation, and lack of knowledge about the disease (Table 2).

**Table 2:** Risk factors associated with microfilaremia of lymphatic filarial parasites

Study (year)	Study area	Sample size	species	Risk factors
Triteeraprap and Songtrus, (1999) [30]	Tak province, Thailand	654	*B.m.*	• Male • History of mosquito bites
Triteeraprapaat al.,(2001) [31]	Narathiwat province, Thailand	2469	*B.m.*	• Male • Older age
Dickson et al., (2018) [21]	Mandalay Region, Myanmar	1014		• Amarapura Township
Wahyuni et al., (2004) [25]	South Sulawesi, Indonesia	583	*B.m.*	• Male, • Older age
Maheudin et al., (1977) [32]	Kepu District, Central Jakarta, Indonesia	614	*W.b.*	• Poorly structured houses • Male
Upadhyayula et al., (2012) [33]	Andhra Pradesh, India	5394		• Older age • Low educational status • Poorly structured houses • Houses close to mud drainage system
Sharma et al., (1999) [34]	Varanasi, India	1850	*W.b.*	• Male
Pani et al., (1991) [35]	Pondichery, South India	41,108	*W.b.*	• Male
Jain et al., (1989) [36]	Kerala, South India	11,604	*B.m.*	• Older age • Male • Low monthly income • Low educational status
Baruah and Rai, (2000) [37]	Uttar Pradesh, India	194	*W.b.*	• Poorly constructed houses
Adhikari et al., (1994) [38]	West Bengal, India	85,974	*W.b.*	• Coal mining areas
Shriram et al., (2002) [39]	Andaman and Nicobar Islands, India	862	*W.b.*	• Male • Older age
Kumar et al., (1994) [40]	Orissa, India	782		• Male • Irregular or never usage of bed nets • “minimum” clothing
Kumar and Chand, 1990 [41]	Orissa, India	13,398	*W.b.*	• Sub coastal villages
Subramanian et al., (2018) [42]	Tamil Nadu, India	603		• Tiled and concrete houses • Proximity of U-drains to a house
Mishra and Bhadoriya, (2009) [43]	Madhya Pradesh, India	78		• Male • Low educational level
Rao et al., (2018) [26]	Southern province, Sri Lanka	528	W.b.	• Male
Byanju and Gupta, (2012) [44]	Dhading District, Nepal	516	*W.b.*	• Male • Older age • Low education level • lack of awareness about the disease, • Poor sanitation • Irregular usage of bed-nets
Mehta PK, Maharjan, (2024) [45]	Lalitpur, Dhading, Bara and Mahottari Districts, Nepal	1722	*W.b.*	• Male

Wb: *Wuchereria bancrofti*; Bm: *Brugia malayi*

Male gender and older age were the most frequently identified risk factors associated with lymphoedema, hydrocele, and elephantiasis. Other contributing elements included low socio-economic status, limited educational attainment, poverty, substandard housing, living in coastal or low-altitude regions, proximity to swamps, pools, or drainage systems, participation in agricultural or outdoor jobs, frequent overnight stays in forests or rice fields, inadequate sanitation, lack of bed net usage, insufficient protective clothing, and low awareness of diseases. Particular local factors, such as residing near livestock, palm plantations, or animal shelters, along with poor waste management, were also emphasized in specific areas (Table 3).

**Table 3:** Risk factors associated with Lymphoedema / Hydrocele /Elephantiasis of lymphatic filarial parasites

Study (year)	Study area	Sample size	species	Risk factors
Williams et al., (2023) [46]	19 endemic districts, Bangladesh	43,678		• severely stunted children, • rural population, • poverty headcount, • primary employment in agriculture, • households without toilet, • households without electricity, • mean temperature
Rauyajin et al., (1995) [47]	Nakorn-srithamarat province, Thailand	1223		• poverty • poor housing conditions • low education level • Night-time fishing at the open swamp • Guarding livestock and crops at night without mosquito netting • Children playing in the swampy forest
Bhumiratana et al., (2002) [29]	Tak Province, Thailand	219	*W.b.*	• Migrants from Myanmar
Dickson et al., (2021) [20]	Mandalay Region, Myanmar	1014	*W.b / B.m.*	• Older age • Residing in Amarapura Township
Dickson et al., (2018) [21]	Mandalay Region, Myanmar	1014		• Older age • Amarapura Township
Odermatt et al., (2018)[48]	North–Eastern provinces of Cambodia,	291		• frequent overnight stays in forests or paddy fields outside the village • Usage of un-impregnated bednets, or no net
Leang et al., (2004) [49]	Mondulkiri, Rattanakiri, Stung Treng and Preah Vihear provinces, Northeastern Cambodia	3500	*W.b / B.m.*	• Male • Older age
Grove et al., (1974) [50]	Catanduanes Province, Philippines	535	*W.b.*	• Male • Older age
Sabesan et al., (2013) [51]	Andhra Pradesh, Bihar, Haryana, Himachal Pradesh, Karnataka, Kerala, Madhya Pradesh, Maharashtra, Odisha, Punjab, Rajasthan, Uttar Pradesh, Uttarakhand, and West Bengal, India	3667 (aged < 15 years)	*W.b / B.m*.	• Coastal and river basins • Low altitude
Sharma et al., (1999) [34]	Varanasi, India	1850	*W.b.*	• Male
Pani et al., (1991) [35]	Pondichery, South India	41,108	*W.b.*	• Male • Older age
Jain et al., (1989) [36]	Kerala, South India	11,604	*B.m.*	• Older age • Low monthly income • Low educational status • Large Family size
Baruah and Rai, (2000) [37]	Uttar Pradesh, India	194	*W.b.*	• Poorly constructed houses
Mutheneni et al., (2016) [52]	Andra Pradesh, India	5133		• Low monthly income People living in tiled house structure • Houses close to mud drainage system
Vanamail and Gunasekaran, (2011) [53]	Pondicherry, South India	531	*W.b.*	• Low educational level • Low awareness and prevention of the disease
Babu et al., (2001) [54]	Orissa, India	5357		• Male • Older age
Shriram et al., (2002) [32]	Andaman and Nicobar Islands, India	862	*W.b.*	• Male • Older age
Sajith et al., (2023) [55]	Kerala, India	72		• Male • Older age
Ramaiah et al., (2000)[56]	Tamil Nadu, India	2108	*W.b.*	ADL: • Male Chronic disease: • Low education
Singh et al., (2024) [57]	Uttar Pradesh, India	233		• Farmers • Usage of public / sharing toilet • Presence of animal shelter or water source inside the house • Low monthly income • Not proper water and solid waste management system in the community
Mehta and Maharjan, (2024) [45]	Lalitpur, Dhading, Bara and Mahottari Districts, Nepal	1722	*W.b.*	• Older age
Edirisinghe, (2017) [58]	Matara District, Sri Lanka	70		• Male • Coastal areas • High population density • Swamp barren land • Methods of garbage disposal
Wayangkau et al., (2025) [59]	Papua, Indonesia	135		• Male • Older age • Low education level • Low monthly income • Not wearing long-sleeved clothing at night
Sapada et al., (2015) [60]	Banyuasin District, Indonesia	132		• Existence of swamp or pool water area • Low educational level • Low knowledge of the disease
Hutagalung et al., (2014) [61]	Agam District, Indonesia	91		• Living near a palm plantation • not using ventilation net or bed net
Maifrizal et al., (2023) [62]	Aceh Province, Indonesia	126		• Animals around the house • Not good role of health workers • Low education
Ikhwan et al., (2016) [63]	Riau Islands Province, Indonesia	98		• Low knowledge of the disease • Not usage of mosquito nets • Presence of swamps • Going out from the home at night
Maheudin et al., (1977) [32]	Kepu District, Central Jakarta, Indonesia	614	*W.b.*	• Poorly structured houses • Male • Years of residency

Wb: *Wuchereria bancrofti*; Bm: *Brugia malayi*

## Discussion

This study revealed that lymphatic filariasis was influenced by socio demographic factors, working activities and the behavioral habits. The observed higher prevalence of lymphatic filariasis (LF) among males and older individuals in Asia underscores the need for targeted interventions. Outdoor occupations at night commonly undertaken by men, such as construction, elevate the risk of brugian and bancroftian filariasis by increasing exposure to mosquito bites, the main mode of transmission for the disease [20, 64].

Adults are more likely than younger people to develop the chronic stage of lymphatic filariasis because lymphedema, elephantiasis, and hydrocele result from a slow, cumulative process. Each infective mosquito bite deposits microscopic larvae that mature into adult worms in the lymphatic system. Over years or decades of repeated exposure in endemic regions, the adults accumulate to a heavier parasite burden, leading to progressive lymphatic vessel damage and fibrosis. Combination of accumulated worm load, chronic immune-mediated injury, and the slow development of lymphatic damage over time causes chronic filariasis predominantly present in adults [65]. Enhancing vector control strategies, including mosquito management and the implementation of protective measures, can aid in reducing the risk of repeated exposure. Health education and community awareness initiatives ought to highlight the cumulative aspect of infection and encourage prompt medical consultation along with self-care practices to avert the advancement of lymphatic damage.

Economically disadvantaged families frequently encounter challenges in obtaining health-related information and preventive resources, such as insecticide-treated mosquito nets and educational outreach programs [66]. They often reside in environments with poor sanitation and substandard housing conditions, which contribute to the spread of disease [67, 68]. Limited access to clean water and adequate sanitation increases exposure to mosquito breeding sites, while overcrowded housing makes it harder to avoid infective bites. Health education and community outreach must be enhanced in low-resource regions to increase awareness regarding disease prevention and early treatment. It is crucial to strengthen and provide consistent funding for health-care services, including mass drug administration programs, to guarantee equitable access to interventions [67, 68]. Furthermore, implementing targeted strategies that address overcrowded living conditions and environmental risk factors can aid in decreasing exposure to infective mosquitoes and alleviate the advancement of disease among vulnerable populations [69].

Individuals with limited education may lack awareness about LF transmission and prevention, leading to lower participation in mass drug administration (MDA) programs and reduced use of protective measures like bed nets. Moreover, low-income households often reside in environments conducive to mosquito breeding, such as areas with poor sanitation and inadequate housing structures [33].

Poorly constructed housing with mud walls, inadequate ventilation, and lack of window screens create favorable resting and breeding conditions for mosquitoes [70]. These conditions, combined with overcrowding, elevate indoor mosquito densities, thereby increasing the likelihood of human-vector contact [20]. Furthermore, inadequate housing is often linked to poor sanitation infrastructure, including open or uncemented drainage systems, which serve as breeding sites for mosquitoes. These findings highlight the critical need for integrated public health interventions that address housing improvements alongside traditional LF control measures [70].

Spending many nights in forests or paddy fields lead to higher contact with mosquito vectors, particularly in rural and agricultural regions where the transmission of diseases is higher [46]. Individuals sleep outside or in inadequately protected shelters in these regions are at a higher risk of being bitten by infected mosquitoes, especially during the night when mosquito activity peaks. To mitigate the increased risk of LF, it is advisable to encourage the adoption of protective measures including insecticide-treated bed nets, repellents, and adequately screened or enclosed sleeping shelters [71]. Health education initiatives should focus on rural and agricultural communities to enhance awareness regarding peak mosquito activity periods and methods to reduce exposure. Moreover, the implementation of community-based vector control strategies in high-risk zones, such as habitat management to decrease mosquito breeding sites, can further diminish transmission [72].

The use of mosquito nets plays a crucial role in mitigating lymphatic filariasis by preventing mosquito bites, particularly during the night when the disease-carrying mosquitoes are most active. Resting under insecticide-treated nets establishes a protective barrier, minimizing exposure to infected mosquitoes and decreasing the likelihood of being bitten. This not only shields individuals but also contributes to diminishing the overall mosquito population and breaking the cycle of disease transmission within the community [73]. The widespread adoption of mosquito nets serves as a straightforward and effective measure that complements other elimination strategies, such as mass drug administration [73].

The implementation of integrated vector control strategies, which includes the use of insecticide-treated bed nets, indoor residual spraying, and environmental management to eliminate mosquito breeding sites, is used to reduce the risk of LF among people living in endemic areas longer time [72, 73]. Public health education should prioritize raising awareness about personal protective measures, such as wearing long-sleeved clothing and utilizing repellents, particularly for individuals residing in or frequently visiting endemic regions [74]. Furthermore, mass drug administration (MDA) programs ought to be enhanced and directed towards long-term residents and high-risk populations to effectively interrupt transmission [75].

There were no research on the risk factors for LF from Yemen, Vietnam, or Lao PDR, most likely for a variety of reasons. These nations might prioritize implementing elimination programs over carrying out and publishing epidemiological studies due to a lack of funding or research capabilities. Priorities have shifted away from researching risk factors in certain countries, such as Vietnam and Lao PDR, where LF has been eliminated or drastically reduced. Furthermore, current research could be published in non-indexed journals or in local languages, which makes it challenging to find in global databases.

There are several limitations to this review. First, we exclusively included English-language publications, which might have resulted in the exclusion of relevant Asian non-English research. Second, it was challenging to compare results across research and might have added heterogeneity due to variations in study design, risk factor definitions, and lymphatic filariasis diagnosis techniques. Finally, because research with significant findings are more likely to be published and indexed in large databases, publication bias might exist.

## Conclusion

Based on the summarized risk factors, it is clear that older age and male gender are consistently linked to a greater vulnerability to lymphatic filariasis, which encompasses circulating filarial antigens, microfilaremia, and chronic conditions. Additional determinants include low socioeconomic status, limited education, inadequate housing, and environmental exposure to mosquito breeding sites (for instance, swamps, drainage systems, and coastal or low-altitude areas). Furthermore, occupational and behavioral risks, such as outdoor employment, frequent overnight stays in forests or fields, and insufficient personal protection measures like bed nets or appropriate clothing, contribute to the risk. In order to reduce the risk of LF and its associated complications, it is imperative that targeted interventions address both environmental and behavioral aspects. Enhancing vector control strategies such as the eradication of mosquito breeding sites, encouraging the regular use of bed nets, and executing indoor residual spraying is vital, especially in low-altitude, coastal, or densely populated regions.

Public health initiatives should emphasize education regarding personal protection, disease awareness, and appropriate sanitation practices, particularly for high-risk demographics. Socioeconomic support programs, aimed at improving housing conditions and ensuring access to clean water and sanitation, can further diminish exposure. Moreover, MDA campaigns must be sustained and customized to effectively reach long-term residents, migrants, and those in high-risk occupational environments. Community involvement and locally tailored strategies, such as providing protective clothing for outdoor workers and managing livestock-related exposure, are essential for achieving lasting reductions in infection rates and preventing conditions such as lymphoedema, hydrocele, and elephantiasis.

## Supplementary Information

Below is the link to the electronic supplementary material.

Supplementary Material 1

## Data Availability

The datasets used and/or analysed during the current study are available from the corresponding author on reasonable request.

## Abbreviations

LF: Lymphatic Filariasis
CFA: Circulatory Filarial Antigens
Wb: *Wuchereria bancrofti*
Bm: *Brugia malayi*
MDA: Mass Drug Administration
WHO: World Health Organization
